# On Meniere's Disease and Its Allied Conditions

**Published:** 1906-11-03

**Authors:** J. Dundas-Grant

**Affiliations:** Surgeon, Central London Throat and Ear Hospital and West End Hospital for Nervous Diseases


### HOSPITAL CLINICS.

### ON MENIERE'S DISEASE AND ITS ALLIED CONDITIONS.

By J. Dundas-Grant, M.D., F.R.C.S.(Eng.), Surgeon, Central London Throat and Ear Hospital and West End Hospital for Nervous Diseases.

(Some Notes of a Lecture delivered at the Polyclinic College.)

Meniere's disease was discussed in a masterly and inspiring lecture delivered here by Dr. Hughlings Jackson in 1903. He dealt with the subject as a neurologist and philosopher. I shall to-day approach it as an aurist and ex-general practitioner.

### A Typical Case.

In this disease the patient is suddenly attacked with giddiness and sickness and vomiting. This is accompanied by great depression, cold sweat, and probably the patient notices a noise in his ears, usually a high-pitched musical sound, and later he discovers that he is deaf in one or both of his ears. From that description there are many variations. In olden times the terrible anxiety occasioned by such an attack was indescribable, and Ménière rendered great service to humanity when he identified the symptomatology of this disease. There are three symptoms — so-called syndrome — vertigo, tinnitus, and deafness. The vertigo is sometimes characterised by being accompanied by severe vomiting rather than by the actual vertiginous sensation. The patient is apt to take the disease for a severe bilious attack. The depression is intense and the tinnitus is sometimes described as being like a railway whistle, the deafness is of the labyrinthine type.

### Relations of Semicircular Canals to Equilibration.

I show you a diagram of the osseous labyrinth of the ear on a highly magnified scale. You observe that there are three semicircular canals, one vertical and nearly sagittal, one vertical and nearly coronal, and one nearly horizontal. They have been compared to the sides and seat of a corner chair (looking in the skull outwards and backwards), and this crude analogy may help you to remember their relative position. I have used the qualifying adverb "nearly" very advisedly, because the posterior canal of one side is in a line with the anterior one of the opposite. Each bony canal has a dilatation, its ampulla. In each of these bony canals there lies a membranous one each again with an ampulla. The membranous canal is about one-third of the diameter of the osseous. When one thinks that the section of the bony canal is $\frac{1}{20}$ in. it may readily be conceived how small the membranous one is $\frac{1}{100}$ to $\frac{1}{60}$in. It contains a fluid the endolymph, which is set in motion when the head is moved, but which lags behind the solid structures both at starting and at stopping. This relative movement of the contained fluid disturbs the hairs of certain cells in the membranous ampullæ and sensations are aroused which help us in judging as to the direction and extent of movements our heads have made. The fibres from these cells run by means of the vestibular nerve to Deiter's nucleus and to roof-nucleus of the cerebellum. Other sensations such as those of the muscular efforts required to maintain the body in equilibrium are conveyed through the direct cerebellar tract and restiform body. Others again from the oculomotor nerves convey sensations indicating the movements of the eyes which take place in our efforts to judge by sight of our relation to surrounding objects.

When the cells in the ampulla of a semicircular canal are quite destroyed a certain amount of guidance is lost; this is a "suppression-phenomena" (*Ausfalls-erscheinung*), and compensation is soon established by means of the remaining factors. When, however, the cells are irritated by disease, increase or decrease of pressure, the sensation of a disturbance of equilibrium is produced, and the patient feels as if he were turning or as if objects were turning round him, the plane of such turning being probably determined by the direction of the canal affected. This is an "irritation-phenomenon" (*Reiz-erscheinung*). The irritation-phenomenon is sometimes followed by the suppression-phenomenon. This sensation of turning or moving in a more or less definite plane is termed "vertigo," and when it is due to an irritation in the labyrinth, aural or labyrinthine vertigo.

The disturbed idea as to position may lead to erroneous attempts at rectification, so that the patient may turn into such attitudes that equilibrium cannot be maintained, and he drops to the ground unless he is able to catch hold of a railing or some firm object, till the irritation subsides and the attack passes off.

Ménière's disease as described by Ménière was attributed by him to an effusion of blood or of a blood-stained fluid into the semicircular canals, such as he found in his first classical case. It was expected that in all cases the same condition would be found post-mortem in the rare event of death occurring. Gêllé found, however, in some well-marked cases that no such effusion was present, that the internal ear appeared normal, and that the main pathological condition was a fixation of the stapes in the oval window and closure of the round one by inflammatory products. In Gêllé's cases there was the typical sclerosis of the middle ear, and the pathology required revision. How can such a condition produce vertigo? Any increase of vascular tension in the labyrinth must produce pressure on the sensitive hair-cells and hence sensations of disturbances of position unless some factors act as safety-valves. Such safety-valves are the oval and round windows, the membranes of which yield so as to neutralise the increase of pressure. If these windows are firmly closed by disease this yielding safety-valve action is absent, and the pressure, not being neutralised, its disturbing effect is produced. Thus it is that in the so-called sclerosis of the middle ear vertiginous attacks may occur and simulate the genuine Ménière disease. We may term the condition sclerosis of the middle ear, with Ménière symptoms or aural vertigo.

You may very reasonably ask why giddiness is not present in all cases of sclerosis of the middle ear. I believe that another factor is necessary in these cases for the production of marked vertigo, and that is a hyper-sensitiveness of the vestibular nerve apparatus, and that this is often the main element in the minor forms of aural vertigo, particularly in cases of neurasthenia.

### Analysis of Cases.

I shall now give briefly the results of observations on a number of cases, for the opportunity of examining which I am mainly indebted to my valued colleagues at the West End Hospital for Diseases of the Nervous System.

Among them I find 61 cases with distinct evidence of involvement of the ear, 15 without—76 in all. The following is the analysis of the cases in which the ear is involved: Labyrinthine effusion, 10; chronic labyrinthitis, 4; labyrinthine vertigo, 1; hereditary labyrinthine syphilis, 2; climateric congestion of labyrinth, 3; gouty labyrinthine effusion, 1; labyrinthine congestion, 6; albuminuric labyrinthitis, 1; fracture of the labyrinth, 2; traumatic effusion into the labyrinth without fracture, 5; syphilis affecting the auditory nerve, 1; suppurative catarrh of the middle ear, 5; chronic non-suppurative inflammation of the middle ear with involvement of the nerve element—that is to say, sclerosis of the middle ear—7; chronic disease of the middle ear with syphilis, 1; residua of suppuration of the middle ear, 1; suppurative inflammation of the middle ear with involvement of the auditory nerve, 1; uncertain, 1. There were nine compound forms, Middle ear disease with epilepsy, whether coincidences or not I cannot say, 2; neurasthenic nerve deafness, 2; hysterical nerve deafness, 1; alcoholic and ocular vertigo and disease of the labyrinth all in the same patient, 1; a doubtful case of hysteria or meningitis, 1; myxodœma, 1; epilepsy along with labyrinthine disease, 1—in all 61 cases.

The following cases simulated Ménière's disease, but were without deafness: Epilepsy, 2; neurasthenia, 5; arterial sclerosis, 3; anæmia, 1; traumatism of the skull without apparent involvement of the auditory nerve or labyrinth, 1; hysteria, 2; myxœdema, 1—15 in all. The vertigo in all these sufficiently resembled that of Ménière's disease, so-called, to require careful distinguishing.

### Scheme of Diagnosis.

The following scheme for the diagnosis of true Ménière's disease and of other conditions simulating it was then discussed. A cardinal symptom of ear disease is deafness, either labyrinthine or obstructive. In nerve deafness the hearing for the tuning-fork on the bone of the skull is diminished, and as a rule the relation between air and bone conduction is unaltered, Rinne's test being positive. The tuning-fork on the vertex is heard best in the good ear, not in the bad one, as is the case in obstructive disease. If the disease is due to the labyrinth you will have loss of hearing greatest for the highest pitched tones as tested with Galton's whistles. The normal ear should hear it up to the mark of 1 millimetre (that is equivalent to the tone produced by a stopped pipe 1 millimetre in length). In nerve deafness it may not be heard below the marks 4, 5, or 6 millimetres, or even more than that. When in nerve deafness loss of hearing is not most marked for the highest pitched tones the trouble is probably central in nature rather than labyrinthine. When the nervous system is exhausted, the sense of hearing along with the other senses is lowered. A sudden occurrence of this intense deafness and vertigo is typical of Ménière's disease proper. When the patient falls, and this is accompanied with a complete loss of hearing, there is probably an effusion of some sort in the labyrinth. If it is a case of concussion or hæmorrhage into the labyrinth the hæmorrhage is very apt to plough up those delicate structures and destroy them altogether.

You may have acute nerve deafness due to acute labyrinthitis arising from some infectious fevers, or chronic labyrinthitis characterised by a certain amount of vertigo, but usually more by deafness. If there is obstructive deafness it may be due to sclerosis of the stapes or disease of the outer wall of the labyrinth. The tuning fork on the vertex in this form will be heard best in the bad ear. Obstructive deafness may be non-suppurative or suppurative deafness. It may be acute or gradual culminating in vertigo. It is vertigo which is labyrinthine vertigo, but it is not due to disease of the labyrinth. With suppuration we may get congestion of the internal ear, accompanied by suppurative symptoms, pressure of granulations upon the stapes or purulent labyrinthitis. Then again there may be no suppuration, merely tympanic tension, causing rigidity of the membranes of the outer wall of the labyrinth and a loss of the safety-valve action already described.

### The Allied Conditions.

What are the allied conditions which are apt to simulate Ménière's disease? Take first those in which there is no loss of consciousness. Vertigo may be the result of epilepsy. If you make out a distinct loss of consciousness and an aura you may as a rule put the case down as being of this nature. If there is a momentary unconsciousness it may be due to syncope arising from cardiac weakness. Sometimes it may cause a slight cough, and then it is termed laryngeal vertigo, though it should properly be called laryngeal syncope.

If there is no unconsciousness, and the vertigo is relieved by closing the eyes, it may be ocular vertigo, neurasthenic, or functional. If it is made worse on closing the eyes it may be due to locomotor ataxy, in which case you look for Argyll Robertson's pupils, test the knee-jerks, and inquire about urinary difficulties. Many of the cases of neurasthenia are relieved on closing the eyes. There is also a functional or mechanical vertigo such as that produced in a swing or roundabout or on the sea. Supposing the vertigo is unaffected by closing the eyes it may depend upon indigestion. Sir William Gowers has said that it is a very rare thing, and that the so-called gastric vertigo is nearly always labyrinthine, 90 per cent. of definite vertigoes being due to trouble in the semi-circular canals.

There are numerous toxic causes, as tobacco and alcoholic and uremia. Pulse tension is sometimes a cause of vertigo associated with early cirrhotic Bright's disease (Brightisme-Auriculaire). Then vertigo may be associated with plethora and other signs of cerebral congestion. This is frequently associated with the menopause. Occasionally injury to the head will produce a lasting tendency to vertigo, even without any apparent injury to the semicircular canals—traumatic vertigo. It is a form of concussion which produces disturbance of the soft parts without actual destruction. Then one must think all the time of the possibility of organic cerebral disease, headache, optic neuritis, ophthalmoplegia, paralysis; unequal pupils and other signs. The locations of cerebral disease which is most likely to be accompanied with vertigo are the cerebellum or the pons and the tegmen of the crus cerebri. Another large group consists of nervous cases (general hypersensitiveness, indicating neurasthenia, and hysteria), often marked by hemianæsthesia and diminution of the field of vision. Then again hemicrania may give rise to vertigo. This shows itself as a unilateral headache, accompanied with scotoma, etc., and finishing up with an attack of vomiting.

### Treatment.

Tide the patient over the attack, keep him at rest, apply warmth to the feet and cold to the head, administer bromide of potassium, and keep the patient in a quiet locality. Among injuries producing labyrinthine disturbance are explosions and exposure to loud noise; hence the need for quietness. There is one fact pretty well established—that the administration of quinine will diminish the sensitiveness of the vestibular nerve. It was originally supposed that it had to be given until it produced absolute deafness. I confess that I had hesitation in carrying it that length, but I found that very small doses of quinine diminished the vertigo considerably and acted as a sedative to the vestibular nerve. Dr. Hughlings Jackson, when lecturing on this subject, stated that such small doses of quinine as half a grain were all that was necessary to keep the vertigo in subjection. I do not say that it will restore the hearing or remove the noises, but it will act favourably on the vertigo in subjection in a great many cases. Supposing that everything of that kind fails and the condition continues to make life a misery, it is justifiable to try evisceration of the semicircular canals. My friends Mr. Lake and Dr. Milligan have carried this out with success. The patient, of course, will be hopelessly deaf in the affected ear, but the vertigo has been checked. If the patient is plethoric there is no doubt that the regular use of aperients may relieve the vertigo; but, on the other hand, there are cases in which the use of an aperient or the loss of a meal will induce the attack. These cases have to be kept in mind, and you must act accordingly. If there is suppurating disease of the middle ear (cholesteatoma) a radical mastoid operation will give relief. Of the other forms simulating Ménière's disease, epilepsy will generally answer to bromides in one form or another, and constitute a great part of the treatment. If the vertigo is due to cardiac syncope the treatment must be regulated accordingly. Vertigo due to pulse tension (auricular Bright's disease) will yield to iodide of potassium and milk and fish diet. In cases of injury to the head the traumatic vertigo is relieved by the administration of small doses (10 minims) of perchloride of mercury. Labyrinthine vertigo will answer to quinine, but if the patient has a fixed idea that quinine upsets him small doses of salicylate of soda will be found effective. In cases of congestion of the internal ear bromide of potassium is very valuable, but it does not produce the same sedative effect on the vestibular nerve as quinine. This may be almost used as a diagnostic agent in difficult cases. cases. In those cases of epilepsy with very slight unconsciousness and well-marked vertigo resembling cases of auricular vertigo where the deafness is not marked and the confusion produced, almost amounts to a loss of consciousness the diagnosis is difficult, but may be cleared up by the use of the drug. Pilocarpine is very useful in cases of congestion of the middle ear, but it should be avoided in weak patients. In cases of nerve deafness in general it must not be administered without the greatest discrimination. Parry and Campbell have both reported obstinate cases which yielded to the antiquated seton in the back of the neck.

